# Reduction of liver metastases outgrowth by tumour antigen-pulsed dendritic cell vaccination

**DOI:** 10.1186/1476-5926-2-S1-S54

**Published:** 2004-01-14

**Authors:** Anna K Mels, Ilvy Mayen, Marjolein van Egmond, Robert HJ Beelen, Sybren Meijer, Cornelia D Richters

**Affiliations:** 1 Surgical Oncology, VU University Medical Centre, Amsterdam, The Netherlands; 2 Molecular Cell Biology, VU University Medical Centre, Amsterdam, The Netherlands

## Introduction

Over 50% of patients diagnosed for primary colorectal carcinoma develop liver metastases. Partial liver resection is the single effective treatment improving dismal survival [1]. Since few patients, however, are candidates for resection, development of therapies that prevent formation of liver metastases is warranted.

Outgrowth of metastases may be reduced or prevented by the action of tumour specific T cells, which are activated in T cell areas of lymph nodes by professional antigen presenting cells (APC). Dendritic cells (DC) are the most potent APC, capable of providing the necessary co-stimulatory signals [2]. DC have been derived *in vitro *from blood monocytes (moDC) for use in clinical trials to generate tumour specific immune responses, and results of vaccination studies in melanoma patients are encouraging [3,4]. However, vaccination for various other cancer types and treatment of established metastases with antigen-pulsed DC has been disappointing.

In the present study we investigated whether vaccination of rats with tumour antigen-pulsed moDC could prevent outgrowth of experimental liver metastases originating from colorectal cancer. Additionally, the therapeutic effect of moDC on existing micrometastases was evaluated.

## Methods

### Animals

Male Wag/Rij rats (Charles-River, Maastricht, The Netherlands) weighing 180–220 g were used in all experiments.

### CC531 Tumour Cells

CC531 is an adenocarcinoma cell line, originating in the colon of Wag/Rij rats exposed to methylazoxymethanol [5]. CC531 cells were cultured in alpha-RPMI (RPMI 1640 medium, supplemented with 2 mM L-glutamine, 10% heat-inactivated foetal calf serum (FCS; Gibco, Bio-Cult, Irvine, Scotland), 50 micromolars beta-mercapto-ethanol, 100 U/ml penicillin and 100 micrograms/ml streptomycin). Cells were detached with trypsin-EDTA solution at 37–C. Viability of cells was assessed by trypan blue exclusion and was always > 95%.

### Generation of MoDC

Peripheral blood mononuclear cells were isolated from heparinized blood by Lymphoprep– (Axis-Shield PoC AS, Oslo, Norway) density gradient centrifugation (d = 1.007 g/ml, 20 min at 800 – g) and placed into culture plates for 2 hours. Subsequently, non-adherent cells were removed and adherent cells were cultured in –-RPMI, containing 5 ng/ml recombinant rat (rr)GM-CSF and rr interleukin (IL)-4 (PharMingen, San Diego, CA, USA) for 7 days. At day 7, non-adherent moDC (moDC-) were isolated by collecting the medium. Viability of cells was always > 95%.

To generate tumour antigen-pulsed moDC (moDC+), a mixture of necrotic and apoptotic CC531 tumour cells was added to moDC- (1:1) at the last day of the 7-day culture. To generate this antigen mixture, CC531 tumour cells were treated with UV-B light, cultured for 24 hours and subsequently subjected to 3 cycles of freezing and thawing.

### Induction of Liver Metastases

Rats underwent small laparotomy under general anaesthesia and a loop of the small intestine was exposed. Under microscopic vision, 1 – 10^6 ^live CC531 tumour cells in 0.5 ml of PBS were inoculated into the mesenteric vein, followed by ligation of the vein, as described earlier [6].

### Vaccination and Treatment Protocol

Rats were immunised subcutaneously with PBS, tumour antigens from 35 – 10^4 ^necrotic CC531 cells, 35 – 10^4 ^moDC- or 35 – 10^4 ^moDC+. In the vaccination protocol, rats were immunised 14 days before inoculation of CC531 cells. Forty days later, rats were sacrificed. In the treatment protocol, rats were inoculated with CC531 tumour cells and immunised 3 days later, when micrometastases had already developed [7]. Rats were sacrificed after an additional 21 days.

Number of tumour nodules was counted macroscopically. Differences were tested using the nonparametric ANOVA with Kruskal-Wallis post-test. Significance was accepted at *P *&lt; 0.05. Results are expressed as mean – SEM.

## Results and Discussion

After vaccination, rats developed 9.1 – 2.7 (PBS, *n *= 12), 15.6 – 5.5 (CC531 tumour antigens, *n *= 7), 2.7 – 2.1 (moDC-, *n *= 7) and 0.8 – 0.4 (moDC+, *n *= 12) tumour nodules. Vaccination with moDC+ reduced outgrowth compared to PBS (*P *&lt; 0.01) and CC531 (*P *&lt; 0.05), indicating that moDC+ vaccination induced effective anti-tumour immune responses. Previously, rat moDC were shown to be capable of migrating to draining lymph nodes and priming T cells *in vivo*, supporting that injected moDC are fully functional *in vivo *[8].

Surprisingly, vaccination with moDC- also induced a reduction in tumour outgrowth compared to PBS (*P *&lt; 0.05). This could have been caused by *in vivo *generation of bystander T cells directed against foreign FCS components, because CC531 cells, moDC- and moDC+ were all cultured in medium containing FCS. Subsequent recognition of FCS components on CC531 cells might have initiated tumour cytotoxicity. To exclude bystander effects in future vaccination experiments, CC531 cells should be cultured in syngeneic Wag/Rij serum.

None of the four treatment protocols reduced outgrowth of micrometastases (PBS: 1.4 – 0.2, *n *= 5; CC531 antigens: 2.8 – 1.2, *n *= 6; moDC-: 1.8 – 0.4, *n *= 6; moDC+: 5.2 – 2.1, *n *= 5). This could be due to the fact that *in vivo *generation of specific T cells after DC vaccination takes several weeks, during which time tumour cells have the opportunity to spread. Additionally, tumour cells have the capacity to suppress immune responses by production of IL-6 [9] and vascular endothelial growth factor (VEGF) [10]. In accordance with our earlier data, it might be valuable to administer adjuvants such as GM-CSF to enhance DC functionality [6].

Our results indicate that tumour antigen-pulsed DC vaccination may be valuable in the prevention of liver metastases formation in primary colorectal carcinoma. However, when micrometastases are already present, DC immunisation seems less effective. Characterisation of effector cells and optimisation of immunisation is therefore essential in future studies.
